# A Stateful Fuzzing Methodology for Security Verification of 5G Core Equipment

**DOI:** 10.3390/s26175415

**Published:** 2026-08-27

**Authors:** Seungjoon Na, Hwankuk Kim

**Affiliations:** 1 Department of Cybersecurity, Kookmin University, Seoul 02707, Republic of Korea; nsj43885@kookmin.ac.kr; 2 Department of Information Security, Cryptography and Mathematics, Kookmin University, Seoul 02707, Republic of Korea

**Keywords:** 5G security, NGAP, AMF, stateful fuzzing, specification-guided security verification, logical security vulnerability

## Abstract

As fifth-generation (5G) networks evolve toward open and software-based architectures, security verification of the NG Application Protocol (NGAP) between the radio access network and the 5G core has become increasingly important. This paper proposes a stateful security verification methodology that combines stateful fuzzing with specification-guided security verification. The methodology derives an Access and Mobility Management Function (AMF) state model, mutation types, and expected behaviors from Third Generation Partnership Project (3GPP) specifications. For each attack scenario, it establishes the required connection state through normal NGAP procedures, injects a mutated message, and compares the observed AMF responses and processing logs with the specification-defined expected behavior. We evaluated four open-source 5G core implementations using 324 attack scenarios per implementation, resulting in 1296 tests. Of these, 877 produced sufficient evidence to interpret the AMF processing outcome, yielding an interpretable outcome rate of 67.7%. The evaluation covered 27 of the 40 uplink NGAP message types and identified 32 specification violations, including 11 security vulnerabilities, none of which caused a core to crash. These results demonstrate the importance of jointly considering connection states and specification-defined behavior in NGAP security verification.

## 1. Introduction

Mobile communication networks have evolved from closed, hardware-centric systems into open and software-driven architectures. Although this transition improves deployment flexibility and scalability, it also introduces heterogeneous components from multiple vendors and expands the set of interfaces that require security verification. A key example is the NG Application Protocol (NGAP), the control-plane protocol between the radio access network (RAN) and the fifth-generation (5G) core [1]. NGAP supports registration, session management, mobility management, and handover procedures between a gNodeB (gNB) and an Access and Mobility Management Function (AMF) [1,2].

The N2 interface over which NGAP operates has traditionally been considered part of a trusted operator-managed domain, and the gNB has therefore been treated as a trusted endpoint. This assumption is increasingly difficult to maintain in open radio access network and private 5G deployments, where gNBs may incorporate hardware and software from multiple vendors and operate in physically distributed environments. Moreover, transport-layer protection can prevent interception and in-transit modification, but it cannot validate malformed or unauthorized NGAP messages generated by a connected gNB. The receiving AMF must therefore verify such messages against the protocol specifications [1,3].

Existing NGAP security studies have largely relied on fuzzing to detect crashes, memory errors, exceptions, and connection failures [4,5,6,7,8]. Structure-aware and stateful techniques improve input validity and allow mutated messages to reach deeper processing paths [7,8,9]. However, reaching a target state does not by itself determine whether the AMF processes a message correctly. An AMF may remain operational while accepting a message that is invalid for the current state, updating a User Equipment (UE) context with unverified information, or omitting a required error response. Detecting such behaviors requires a test oracle based on specification-defined security properties and expected outcomes [10,11,12,13,14].

This paper proposes a stateful NGAP security verification methodology that combines stateful fuzzing with specification-guided security verification. The methodology derives an AMF connection-state model, mutation types, and expected behaviors from 3GPP specifications. For each attack scenario, it establishes the required state through normal NGAP procedures, transmits a mutated message, and compares the observed AMF responses and processing logs with the specification-defined expected behavior. This process enables the detection of logical security vulnerabilities that arise without crashes or other explicit execution failures.

We evaluated the proposed methodology on four open-source 5G core implementations by applying 324 attack scenarios to each implementation, resulting in 1296 tests in total. The evaluation covered all five defined connection states and 27 of the 40 uplink NGAP message types, identifying 32 specification violations, including 11 security vulnerabilities. The vulnerabilities were responsibly disclosed and subsequently patched.

The main contributions of this paper are as follows:

- State-based NGAP attack scenarios: We derive an AMF connection-state model from the dependencies among NGAP procedures and construct attack scenarios by combining applicable messages, mutation types, and specification-defined expected behaviors.
- Stateful security verification methodology: We combine stateful fuzzing, which establishes the required connection state before injecting a mutated message, with specification-guided security verification, which evaluates the resulting behavior against the protocol specifications.
- Evaluation on open-source 5G cores: We evaluate four open-source 5G core implementations and show that the proposed methodology exercises deeper connection states and a broader set of uplink NGAP messages than the existing fuzzing tools used in the comparison. The evaluation also identifies logical vulnerabilities that do not cause the tested cores to crash.

## 2. Background and Related Work

### 2.1. Background

#### 2.1.1. 5G Architecture

The standalone 5G architecture follows a service-based architecture in which the control and user planes are implemented as separate network functions [2]. The control plane includes the AMF, Session Management Function (SMF), Authentication Server Function, and Unified Data Management, whereas the User Plane Function (UPF) forwards user traffic. Among these functions, the AMF serves as the control-plane entry point between the RAN and the 5G core and manages procedures such as user registration and mobility management.

A gNB connects to the AMF through the N2 interface, over which NGAP carries control-plane signaling. NGAP messages received from the gNB are processed by the AMF and may trigger subsequent procedures, including Non-Access Stratum (NAS) transport, session establishment, and mobility management. Because these processing outcomes can also affect other core functions, such as the SMF and UPF, NGAP messages represent externally supplied control-plane inputs to the AMF and therefore constitute an important security verification target [15,16].

**Figure 1:**
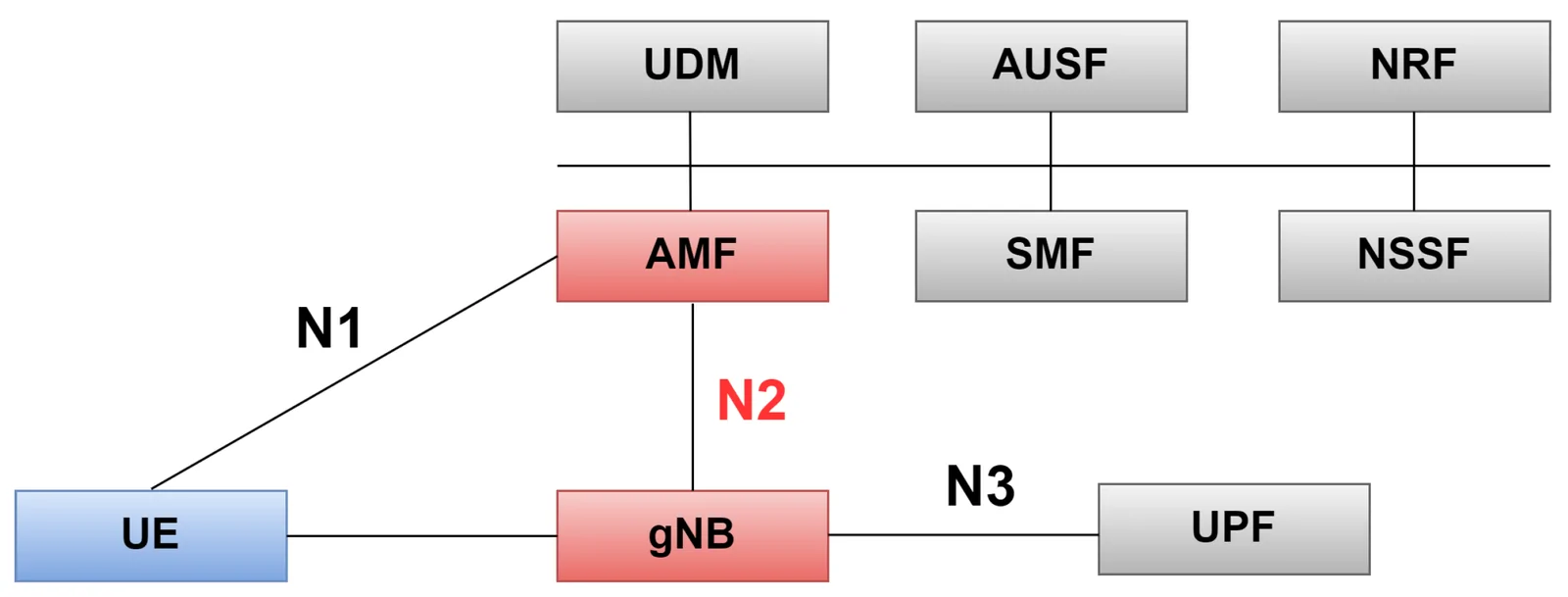
The 5G core architecture and the N2 interface between the gNB and AMF.

The 5G core architecture and the N2 interface between the gNB and AMF are illustrated in Figure 1. This study focuses on NGAP messages transmitted from the gNB to the AMF.

#### 2.1.2. NG Application Protocol

NGAP is specified in 3GPP TS 38.413 and operates over the Stream Control Transmission Protocol (SCTP), with messages encoded using Abstract Syntax Notation One (ASN.1) Packed Encoding Rules (PER) [1]. NGAP defines Elementary Procedures for functions such as user registration, NAS transport [17], Protocol Data Unit (PDU) session resource control, UE context management, and handover.

These procedures are not executed independently. The gNB and AMF first establish an NG connection through the NG Setup procedure. An Initial UE Message then establishes a UE-associated logical NG connection, and the Initial Context Setup procedure establishes the corresponding UE context. Subsequent PDU session and handover procedures depend on the connections and context created by these preceding procedures. Consequently, the current connection state determines both whether a procedure can be executed and whether a received message is valid.

The AMF must therefore validate not only the syntax of an NGAP message but also its consistency with the current connection state. UE-associated messages are bound to a UE context through the AMF-UE-NGAP-ID and RAN-UE-NGAP-ID, requiring the AMF to verify that the identifiers correspond to the correct UE-associated logical NG connection. In addition, procedures such as PDU session management and handover must not be processed before the required UE context has been established. Thus, a message with a valid ASN.1 structure and valid Information Element (IE) formats may still be invalid if it is received in an inappropriate connection state.

### 2.2. Related Work

#### 2.2.1. Fuzzing of 5G Control-Plane Protocols

Security testing of 5G control-plane protocols has primarily relied on fuzzing to assess the robustness of core implementations. Early approaches used replay-based techniques in which captured control-plane traffic was reused as test input. 5Greplay [4], for example, replayed captured NGAP and NAS traffic and mutated selected fields to analyze their impact on AMF availability. Although replaying real traffic facilitates the reproduction of valid procedures, the test space remains limited to previously captured messages.

Mutation-based fuzzing was subsequently introduced to increase input diversity. AMFuzz [5] mutated fields in valid NGAP messages, while Hu et al. [6] selected mutation targets according to field-specific weights. These approaches generate diverse inputs, but mutations that do not preserve the ASN.1 structure are often rejected during decoding and therefore fail to exercise the NGAP processing logic.

Structure-aware fuzzing addresses this limitation by preserving syntactic validity. ASNFuzzGen, the ASN.1-based fuzzing component introduced in RANsacked [9], generated mutated inputs while preserving the ASN.1 structure, thereby increasing the likelihood that the inputs would reach post-decoding processing stages. However, structural validity alone does not account for whether a message is permitted in the current connection state.

Later studies incorporated procedural dependencies through stateful fuzzing. Hsu et al. [7] used a proxy-based fuzzer-in-the-middle architecture to mutate NGAP and NAS messages while preserving ongoing procedures. 5GC-Fuzz [8] constructed a protocol state machine from 3GPP specifications and used it to generate fuzzing paths that reached deeper states. These approaches improve state exploration, but their detection criteria primarily focus on crashes or abnormal state transitions rather than whether the observed behavior conforms to specification-defined expectations.

#### 2.2.2. Specification-Guided Security Verification

Specification-guided security verification evaluates whether protocol behavior conforms to security properties and expected outcomes derived from technical specifications. This approach can identify logical violations that do not manifest as crashes, such as the acceptance of prohibited messages or the omission of required responses.

Silveira et al. [18] developed my5G Tester to evaluate the conformance of NAS and NGAP procedures in open-source 5G cores based on 3GPP specifications. However, its abnormal NGAP input testing considered only an NG Setup Request containing unsupported network parameters and did not systematically evaluate diverse security-requirement-violating NGAP inputs across different connection states.

Early studies primarily focused on the Radio Resource Control (RRC) and NAS protocols in Long-Term Evolution (LTE) and 5G systems. LTEFuzz [10] dynamically tested LTE control-plane procedures using specification-derived security properties, while 5GReasoner [11] formally modeled 5G RRC and NAS procedures and verified security and privacy properties through model checking. DoLTEst [12] further incorporated connection states into specification-driven negative testing by generating invalid downlink messages and evaluating device behavior against state-dependent expected outcomes.

Subsequent studies extended this perspective to state transitions and context consistency in core networks. CITesting [13] examined context-integrity violations across LTE core procedure chains and UE connection states. CoreCrisis [14] combined state-aware fuzzing with specification-derived compliance properties for NAS procedures in 5G core implementations. However, NGAP introduces different state-dependent conditions involving the NG connection, UE-associated logical NG connection, AMF-side UE context, UE identifier binding, and procedure-specific dependencies such as handover. Therefore, applying a NAS-oriented state model directly to NGAP is insufficient; NGAP-specific connection and procedural conditions must be separately abstracted and linked to the validity and expected processing behavior of each NGAP message.

#### 2.2.3. Research Gap

As summarized in Table 1, prior studies have developed along two complementary directions. Fuzzing techniques improve input diversity, structural validity, and state exploration, whereas specification-guided approaches evaluate whether an implementation behaves as required by the relevant standards.

Applying these approaches independently, however, is insufficient for NGAP security verification. Because the validity and expected processing outcome of an NGAP message depend on the connection state established by preceding procedures, security testing must both reach the required state and evaluate the resulting behavior against specification-defined expectations.

This study addresses this gap by linking NGAP-specific state abstraction, state-dependent scenario construction, and specification-defined behavioral verification. Rather than treating the connection state only as a means of reaching deeper execution paths, the proposed methodology uses the state as a condition for determining whether a given NGAP procedure is valid. The resulting state–message–mutation combinations are then evaluated against specification-defined expected behaviors to identify logical violations that may occur without crashes or other explicit execution failures.

To further characterize the methodological differences among NGAP fuzzing studies, Table 2 compares their test construction, use of state or context information, and result-evaluation criteria.

The comparison shows that existing NGAP fuzzing studies primarily differ in how test inputs are generated and how protocol state is incorporated into the testing process. Replay- and mutation-based approaches generate diverse inputs from captured or valid messages, while structure-aware and state-guided approaches improve input validity or enable testing at deeper protocol states. In contrast, the proposed methodology uses NGAP connection and UE-context states not only to reach a target execution condition but also as part of the test condition itself, and it also evaluates the resulting AMF behavior against specification-defined expectations. Accordingly, the proposed methodology is positioned as a state-dependent security verification approach that complements conventional fuzzing objectives focused on input generation, crash detection, or abnormal execution behavior.

## 3. Proposed Methodology

### 3.1. Security Threat Modeling

This section defines the scope, attacker assumptions, and attack objectives for NGAP procedures between the gNB and AMF. Based on these elements, we derive multiple state-based attack scenarios.

Scope. The analysis focuses on NGAP messages transmitted from the gNB to the AMF and the corresponding NGAP processing logic of the AMF. We examine whether the AMF validates message structures and values, UE identifiers, connection states, and UE contexts. We then compare the observed processing outcomes with the expected behavior defined in the specifications. The radio interface, user-plane protocols, direct mutation of NAS messages, and denial-of-service attacks based on high-volume traffic are outside the scope of this study.

Attacker assumptions. The attacker is assumed to control a gNB-side entity with N2 access to the AMF and sufficient gNB-level protocol functionality to establish an SCTP connection and execute normal NGAP procedures. This threat model does not assume an arbitrary unauthenticated remote attacker. The attacker can establish an NG connection, a UE-associated logical NG connection, and a UE context through normal procedures, and can subsequently mutate IEs, UE identifiers, and security-related information or transmit messages that are not permitted in the current connection state. However, the attacker has no direct access to the internal code, memory, or database of the AMF.

Attack objectives. The attack objective is to cause the AMF to process a mutated NGAP message without performing the validation or producing the response required by the specifications. Such behavior may violate the consistency between UE identifiers and connection states, trigger subsequent procedures without the required UE context or preceding procedures, or incorporate security-related information without comparing it with the existing context.

#### 3.1.1. Definition of the AMF State Model

The validity of an NGAP message depends not only on the message itself but also on the connection state in which it is received. A state model that represents AMF connection states is therefore required to derive state-based attack scenarios.

**Figure 2:**
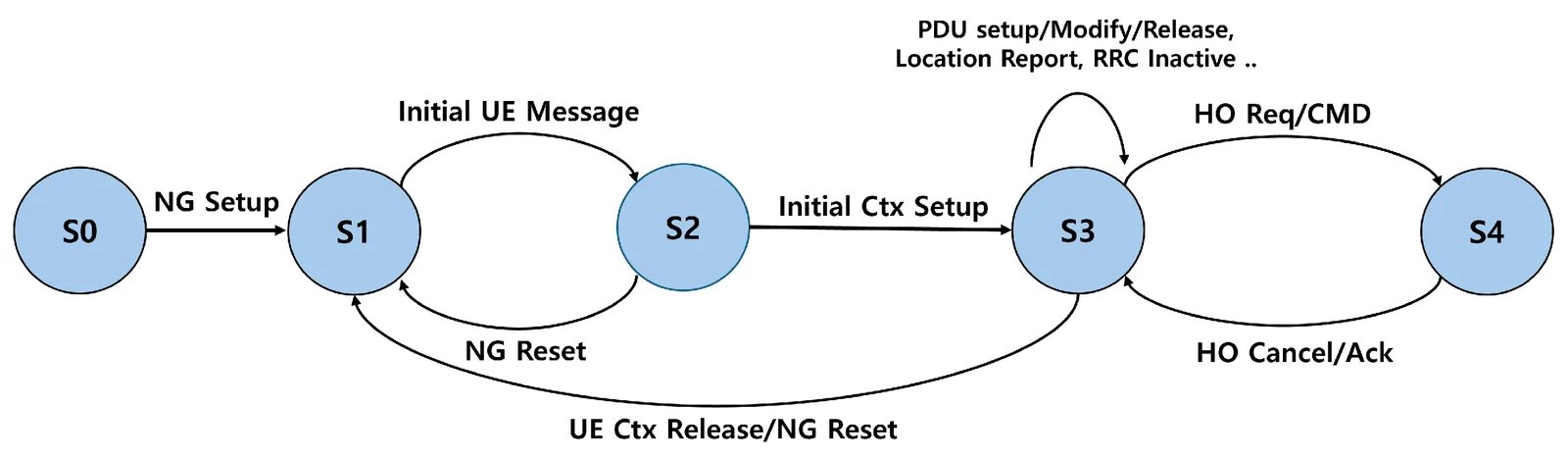
AMF connection-state model derived from NGAP procedure dependencies.

The 3GPP specifications do not explicitly define a standalone state model for AMF NGAP connection states. TS 24.501 defines 5G mobility-management states from the UE perspective, whereas TS 38.413 specifies NGAP procedures and their execution conditions [1,17]. We therefore derive the AMF state model by analyzing the dependencies among UE-associated NGAP procedures defined in TS 38.413. The resulting state model is presented in Figure 2.

TS 38.413 classifies NGAP procedures as UE-associated or non-UE-associated [1]. Non-UE-associated procedures do not depend on a specific UE context, whereas UE-associated procedures rely on connections and UE contexts established through preceding procedures. We therefore analyze the relationships between preceding and subsequent procedures from the perspective of UE-associated procedures.

The analysis shows that UE-associated procedures rely on three sequential layers: the NG connection, the UE-associated logical NG connection, and the UE context. Completion of the NG Setup procedure establishes the NG connection between the gNB and AMF. The Initial UE Message then establishes a UE-associated logical NG connection, and completion of the Initial Context Setup procedure establishes the UE context. Most UE-associated procedures are executed only after these layers have been established.

Based on these dependencies, we define the initial state as S0, the NG-connected state as S1, the UE-associated logical NG connection state as S2, and the UE-context-established state as S3. The states are sequentially dependent, such that each state beyond S0 requires the successful completion of the procedures associated with the preceding state.

The purpose of this abstraction is not merely to represent the progression of NGAP procedures. Each state captures the connection and UE-context conditions that determine whether a particular NGAP procedure is valid in that state, and is therefore used as a state-dependent verification condition when constructing attack scenarios.

Procedures executed after UE context establishment are further divided into general UE-associated procedures and handover-related procedures. Procedures such as PDU Session Resource Setup are executed while retaining the UE-context-established state. By contrast, Handover Preparation creates a separate procedural state that must exist before subsequent handover messages, including Handover Cancel and Handover Notify, can be processed. We therefore define the handover-in-progress state as S4.

The state model includes not only forward transitions established through normal UE-associated NGAP procedures but also transitions that release, reset, or terminate existing connection and procedural conditions. For example, UE Context Release removes the established UE context, NG Reset resets the affected UE-associated logical NG connections and related UE contexts through non-UE-associated signalling, and Handover Cancel terminates an ongoing handover procedure. These transitions allow the model to represent release, reset, and rollback behavior in addition to the forward progression from S0 to S4.

The state model is not intended to represent every internal AMF state or all possible abnormal recovery paths. Instead, it abstracts the connection, UE-context, and procedural conditions required to determine whether an NGAP message is valid in a given state. The model is subsequently used to identify the NGAP messages applicable to each state and to generate state-based attack scenarios by applying the defined mutation types.

Table 3 summarizes representative NGAP messages that can be transmitted in each state. These state-specific message sets characterize the states and provide the reference inputs used to generate mutated messages.

#### 3.1.2. Derivation of Attack Scenarios

Attack scenarios are derived from the AMF state model, NGAP error-handling rules, and security requirements defined in the specifications. We first define the mutation types applicable to NGAP messages and combine them with the messages available in each connection state. The collected responses and processing logs are then compared with the expected behavior to evaluate compliance with the specifications and identify specification violations.

TS 38.413 [1] classifies NGAP errors as Transfer Syntax Errors, Abstract Syntax Errors, and Logical Errors and specifies the required handling according to the error type and IE criticality. TS 33.501 [3] defines protection and verification requirements for security-related information, including UE security capabilities. Based on these requirements, we define six mutation types: mandatory IE omission, syntactic mutation, semantic mutation, identifier mismatch, state violation, and security capability mutation. Table 4 summarizes their application methods, specification bases, and expected AMF behaviors.

Mandatory IE omission and syntactic mutation violate structural validity. Semantic mutation and identifier mismatch violate procedural or UE-context consistency. A state violation does not modify the message itself; instead, it transmits the message in a connection state in which the procedure is not permitted. The required target state must therefore be established through preceding NGAP procedures. Security capability mutation modifies the received UE security capabilities to determine whether the AMF validates them against the previously stored values.

**Figure 3:**
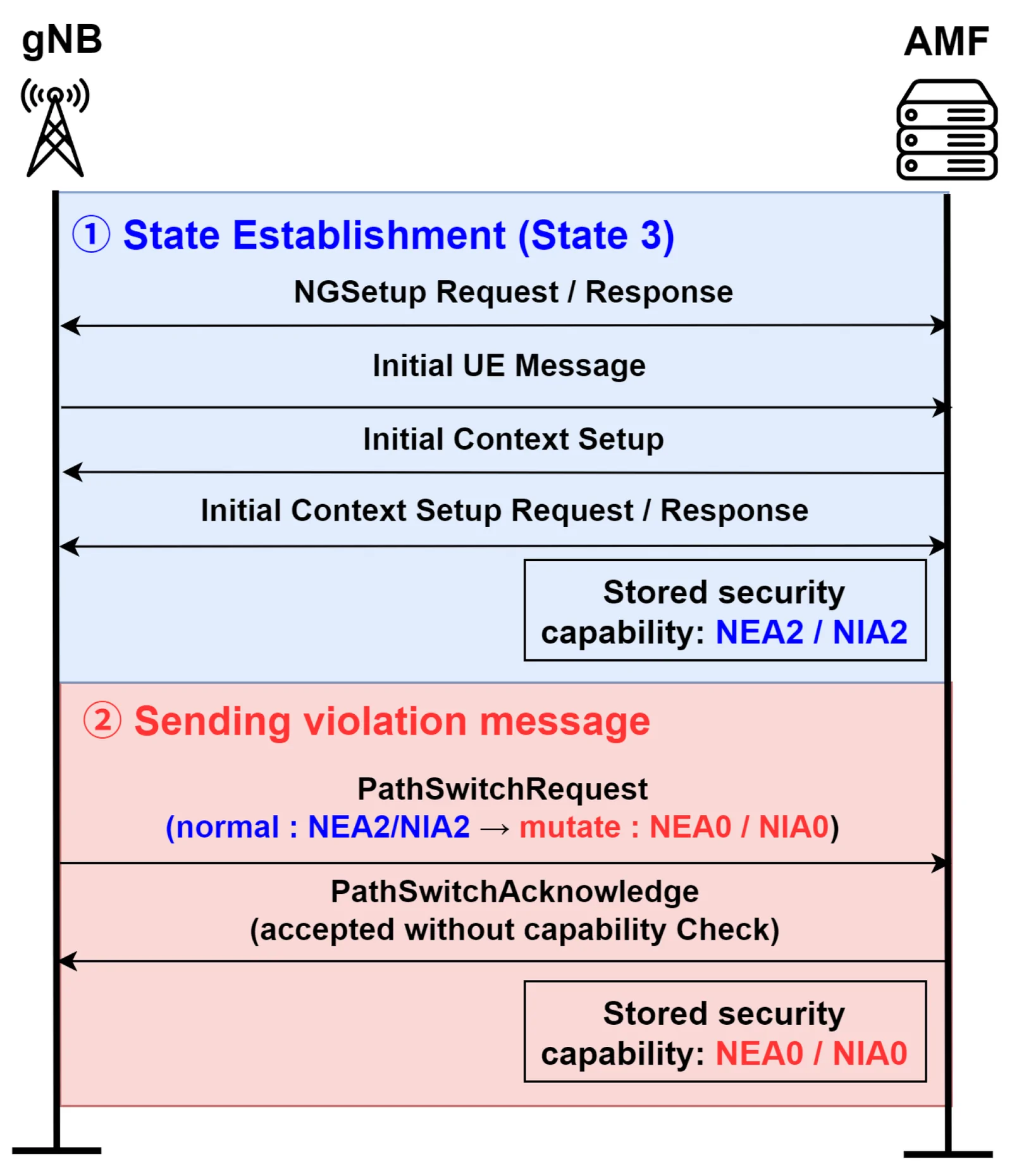
Security capability mutation scenario.

**Figure 4:**
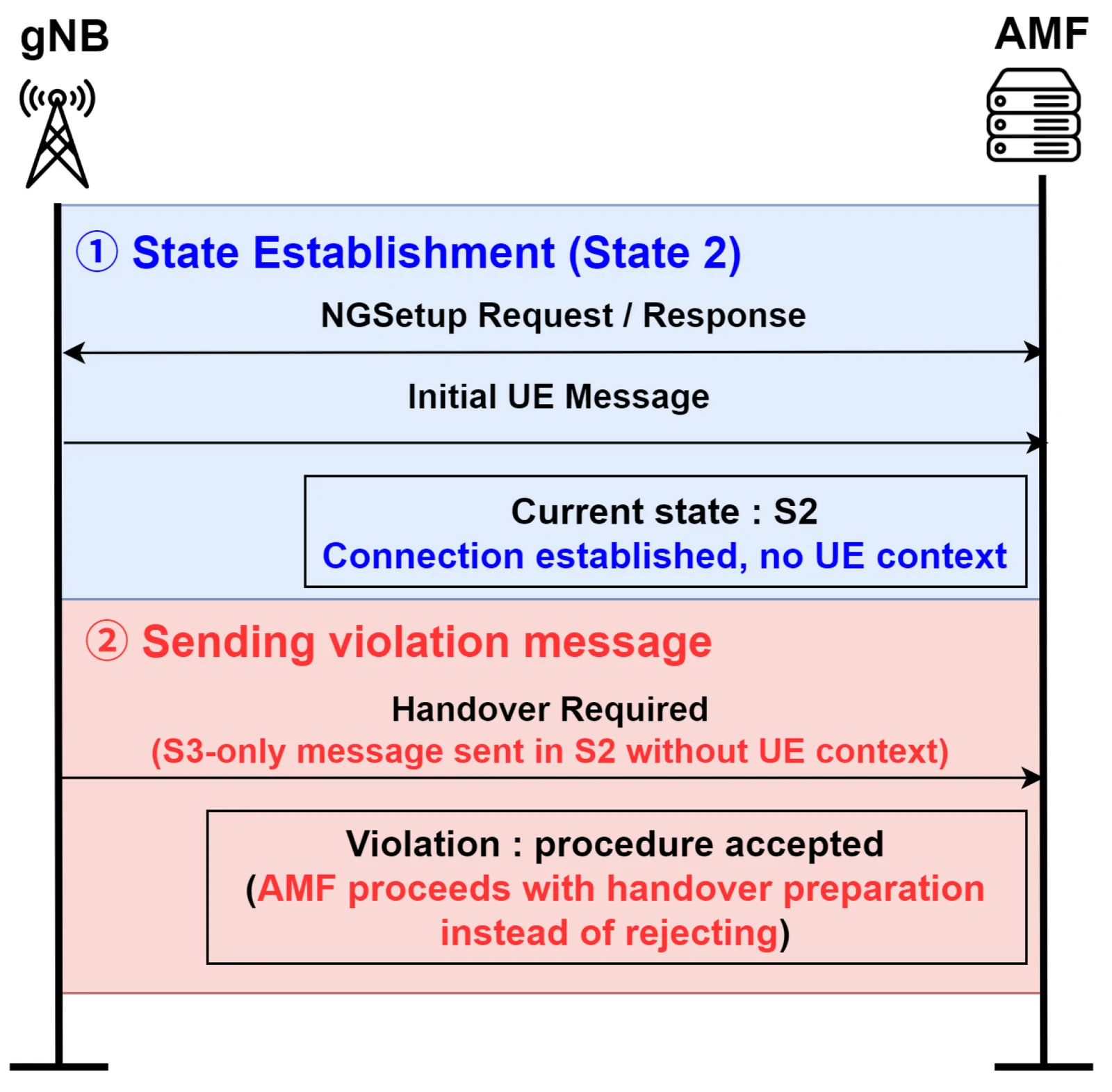
UE context validation bypass scenario.

**Figure 5:**
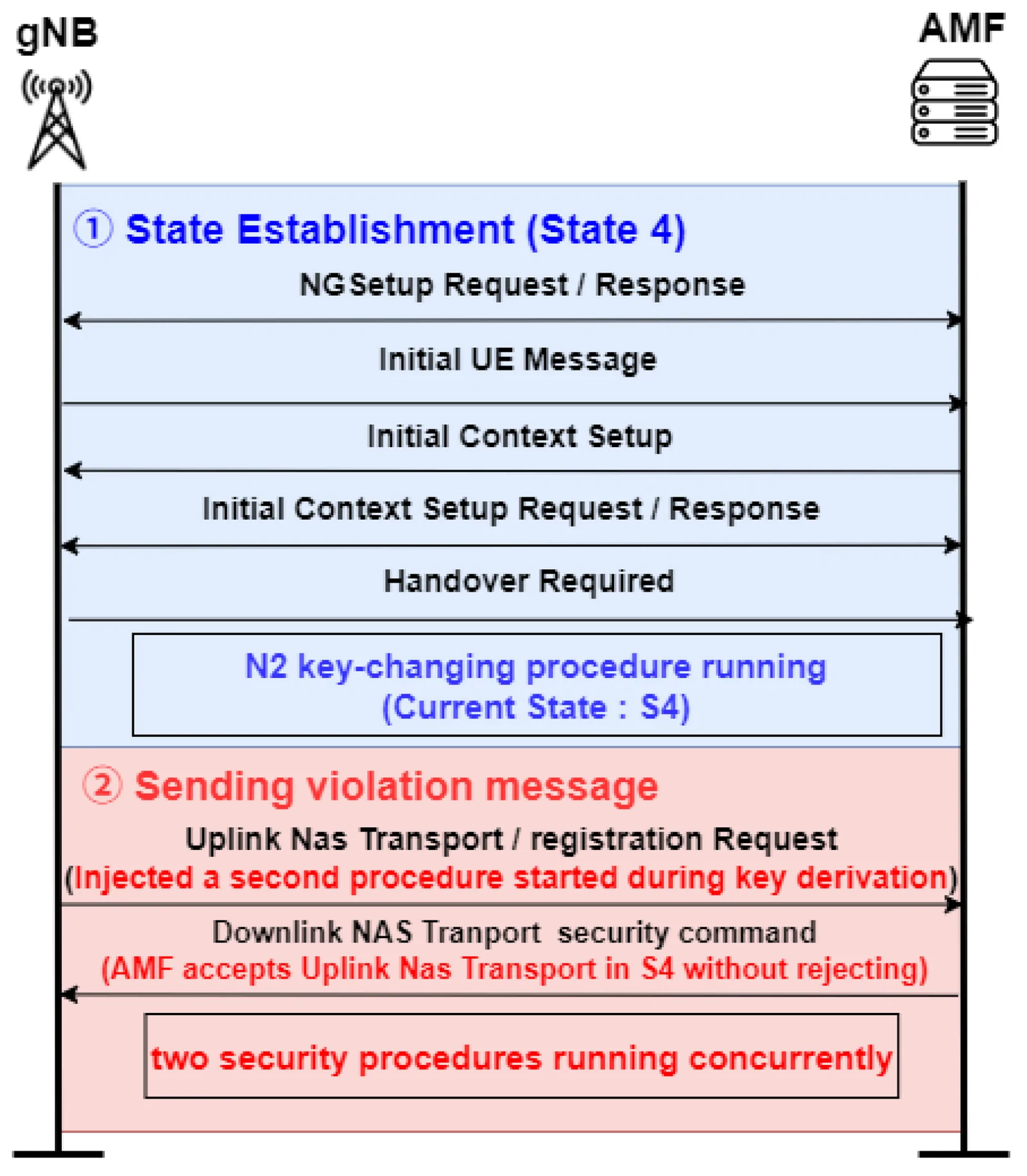
State violation caused by concurrent procedure execution during handover preparation.

Each attack scenario explicitly combines a target connection state, an NGAP message applicable to that state, a mutation method, and a specification-defined expected behavior. In this construction, the connection state serves as part of the test condition rather than merely as a prerequisite for message transmission, allowing the same mutation principle to be evaluated under different connection and UE-context conditions. The representative scenarios shown in Figure 3, Figure 4 and Figure 5 illustrate these state-dependent combinations. Each scenario is evaluated based on an error response, the execution of a subsequent procedure, or a change in the relevant AMF context.

- Security capability mutation scenario.

This scenario verifies that UE security capabilities stored by the AMF cannot be arbitrarily changed during a subsequent NGAP procedure. A normal registration procedure is first executed to establish S3, during which the AMF stores the UE security capabilities. The UE Security Capabilities IE in a Path Switch Request is then replaced with values that are inconsistent with the capabilities previously stored for the UE.

The AMF must verify the received capabilities against the previously stored values and must not overwrite the stored security context with inconsistent values. If the AMF performs such an unverified update, the result is classified as a violation of the security requirements. This scenario is therefore evaluated by examining changes to the stored security capabilities rather than by observing whether an error response is returned. The complete procedure is illustrated in Figure 3.

- UE context validation bypass scenario.

This scenario verifies whether the AMF confirms the existence of the required UE context before processing a UE-associated procedure. State S2 is first established, in which the NG connection and UE-associated logical NG connection exist, but the UE context has not yet been established.

A Handover Required message, which assumes an established UE context, is then transmitted. The AMF is expected to validate the UE identifiers and the current connection state and reject the procedure if the required UE context does not exist. If the AMF accepts the message without validating the context and proceeds with a subsequent message such as Handover Request, the result is classified as a state violation in which handover is initiated without the required context-establishment procedure. The scenario is evaluated using the returned error response and the presence of subsequent NGAP messages. The complete procedure is illustrated in Figure 4.

- State violation caused by concurrent procedure execution.

This state-violation scenario verifies whether the AMF prevents an unrelated UE-associated procedure from being initiated while handover-related key derivation is in progress.

A Handover Required message is first transmitted to initiate the handover preparation procedure and establish S4. While the AMF is processing the handover procedure and deriving the required security keys, an unmodified Uplink NAS Transport message is transmitted through the same UE-associated logical NG connection.

The AMF is expected to reject or defer the Uplink NAS Transport message because that procedure is not permitted while the handover preparation procedure is in progress in S4. If the AMF accepts the message and initiates the NAS transport procedure concurrently with the ongoing handover procedure, the result is classified as a state-violation vulnerability. The outcome is determined by examining error responses, subsequent NGAP messages, and relevant processing logs. The complete procedure is illustrated in Figure 5.

### 3.2. Security Verification Workflow

**Figure 6:**
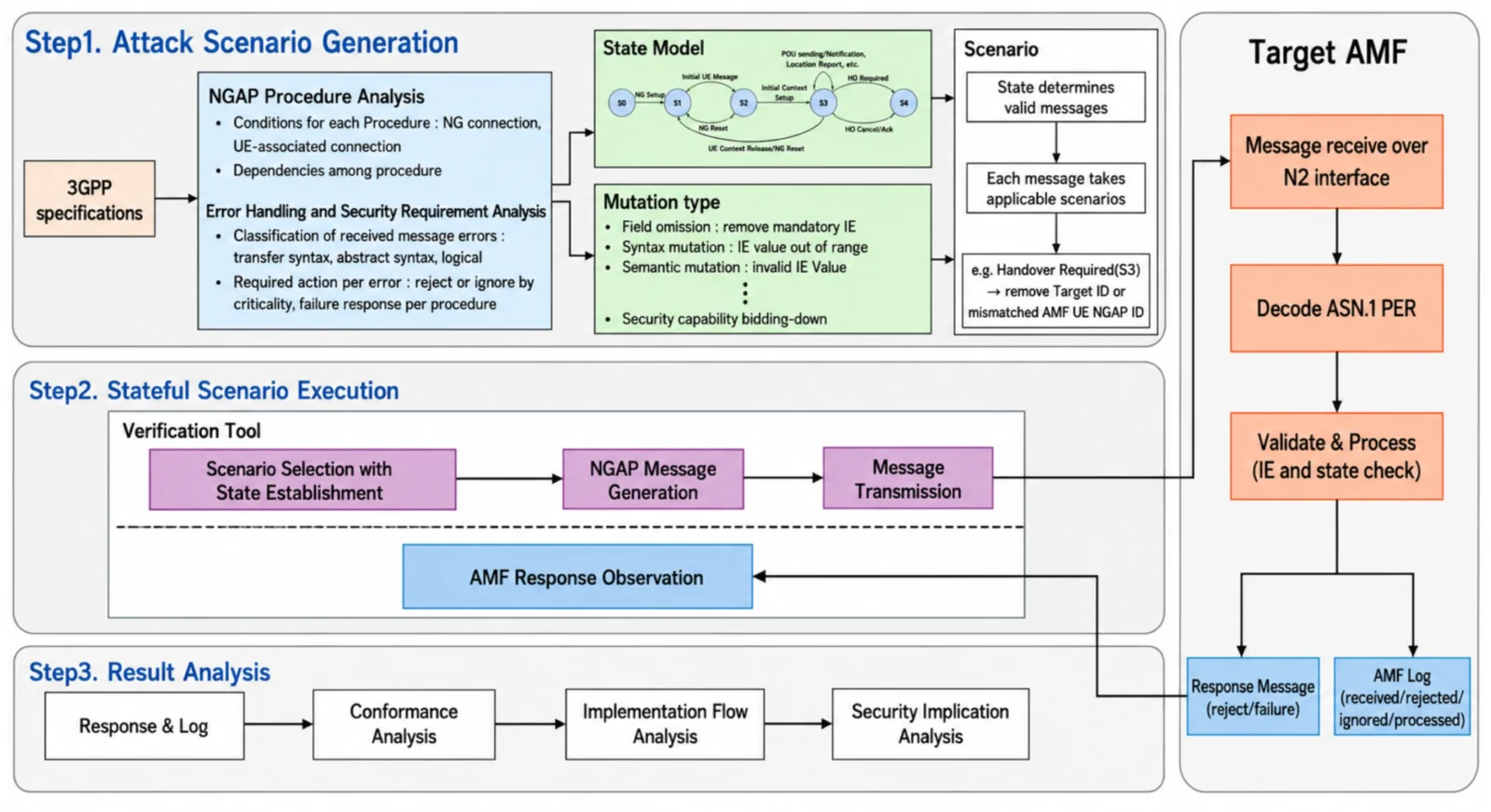
Overview of the proposed stateful security verification workflow.

This section describes the stateful security verification workflow based on the attack scenarios derived in Section 3.1.2. As shown in Figure 6, the workflow consists of scenario selection, mutated-message generation and transmission, and response and log collection.

- Scenario selection.

The verification tool determines whether the current connection state matches the state required by the selected scenario. If not, it follows the transition path defined in the state model and sequentially executes normal NGAP procedures until the target state is reached.

The tool verifies each transition using the AMF response and tracks the completed procedures and established connection information. If the required state cannot be reached, the mutated message is not transmitted, and the scenario is recorded as a state-establishment failure.

State-establishment procedures and mutated-message transmission are executed consecutively through the same gNB instance. This preserves the SCTP connection, UE context, and NGAP identifiers created during state establishment and ensures that the mutated message is processed in the intended connection state.

- Mutated-message generation and transmission.

The selected NGAP message is generated and mutated according to the attack scenario. The mutated message is constructed using the NGAP message structures provided by UERANSIM, and the required IEs or field values are modified according to the selected mutation type. Message structures and IEs that are not mutation targets retain their valid values.

Depending on the scenario, the tool removes a mandatory IE, modifies an IE value, replaces an NGAP identifier, mutates the UE security capabilities, or transmits a message in a prohibited connection state. The resulting message is encoded using the existing ASN.1 PER encoding process and transmitted to the target AMF over the SCTP connection maintained during state establishment.

- Response and log collection.

The verification tool collects NGAP responses and processing logs to determine how the AMF processes the mutated message. NGAP responses are captured in the test environment, whereas processing logs are collected separately from the target AMF. Responses are classified as an Error Indication, a procedure-specific failure message, a normal subsequent message, or no response. When an Error Indication or failure message is returned, the corresponding Cause and Criticality Diagnostics are also recorded. If a normal NGAP procedure continues after transmission of the mutated message, the subsequent messages and procedure information are recorded as well.

For some scenarios, the processing outcome cannot be determined from NGAP responses alone. The processing logs are therefore analyzed to confirm whether the message was processed and whether a subsequent procedure was executed. The collected responses and logs are used in the experimental analysis.

## 4. Experimental Design

This section describes the experimental setup and evaluation procedure. The evaluation consisted of two experiments. The first experiment applied the attack scenarios derived in Section 3.1.2 to verify the security of NGAP processing in each AMF. The second experiment compared the proposed methodology with existing NGAP testing tools in terms of test-case count, message coverage, execution time, and the range of connection states that could be tested. The target cores, execution environment, experimental procedures, and evaluation metrics are described in the following subsections.

### 4.1. Experimental Environment

Target cores. The evaluation targeted four open-source 5G cores: ella-core v1.8.1 [19], Open5GS v2.7.6 [20], free5GC v3.3.0 [21], and OpenAirInterface (OAI) v2.0.1 [22]. All four cores support the NGAP procedures defined in TS 38.413 and are implemented using independent codebases written in Go or C/C++. This configuration allows the same attack scenarios to be applied to different core implementations and enables a comparative analysis of their NGAP processing outcomes.

Execution environment. All experiments were conducted on an Ubuntu 22.04.5 LTS host equipped with 8 vCPUs and 16 GB of RAM. Each core was executed separately on the same host. A gNB and UE based on UERANSIM v3.2.7 [23] were used to execute NGAP procedures, establish the connection states required by the attack scenarios, and transmit the mutated NGAP messages.

Before each scenario, the target core was reset to remove the UE contexts and connection states created by the preceding test. This procedure ensured that the same initial conditions were applied to all cores and prevented previous scenarios from affecting subsequent tests. Table 5 summarizes the target cores and execution environment.

### 4.2. Security Verification of 5G AMFs

Objective. The first experiment evaluated the security of NGAP processing in the AMFs of the four target cores using the attack scenarios derived in Section 3.1.2. Each attack scenario served as an independent test unit for determining whether the AMF exhibited the behavior required by the specifications. The same scenario set was applied to all four cores so that their processing outcomes could be compared under identical test conditions.

Experimental procedure. For each attack scenario, the required AMF state was established through the normal state-establishment path defined by the preceding NGAP procedures, after which the mutated NGAP message was transmitted in the established state. Although the state model also includes release, reset, and rollback transitions, these transitions were not used as alternative state-establishment paths in the experiments. Instead, the normal establishment path was used consistently to reproduce the same target-state conditions across scenarios and core implementations. After each scenario, the connection was released and the UE context was reset before the next scenario was executed.

Test-case configuration. The same 324 attack scenarios were applied to each core, resulting in 1296 tests across the four target cores. The scenarios were organized according to mutation type and AMF connection state. Table 6 and Table 7 summarize the number and proportion of scenarios in each category.

The execution of each scenario was tracked through four stages: scenario configuration, target-state establishment, mutated-message transmission, and outcome interpretation. The 324 configured scenarios were applied independently to each of the four cores, resulting in 1296 scenario executions. A mutated message was transmitted only when the required target state had been successfully established; otherwise, the execution was recorded as a state-establishment failure. After transmission, an outcome was classified as interpretable only when an explicit AMF response or sufficient processing evidence was available to determine how the input was handled. Accordingly, the denominator of the interpretable outcome rate is the full set of 1296 scenario executions, whereas the numerator includes only the executions for which the final AMF processing outcome could be determined.

Analysis method. For each interpretable scenario, the observed AMF processing outcome was compared with the expected behavior derived from the applicable 3GPP requirement. A case was classified as a specification violation when the observed behavior deviated from the specification-defined expectation. A specification violation was further classified as a security vulnerability only when the observed behavior caused a security-relevant effect on the UE context, identifier binding, security-related information, or subsequent NGAP procedure. The classification was based on observable evidence, including the AMF response, processing logs, changes in the relevant UE context, and subsequent NGAP messages, where applicable. The same evidence criteria were applied consistently across the four core implementations.

### 4.3. Comparison Setup for Existing NGAP Fuzzing Tools

Objective. The second experiment compared the proposed methodology with existing NGAP fuzzing tools in terms of test-case count, message coverage, average execution time per test, and the range of AMF connection states that could be tested.

Comparison tools and test cases. 5Greplay [4] and ASNFuzzGen [9] were selected for direct experimental comparison because their source code and execution procedures are publicly available and could be reproduced in the same experimental environment. Other NGAP fuzzing studies [5,6,7,8] were not included in the direct experiment because a publicly available implementation or sufficiently reproducible execution procedure could not be obtained for an equivalent evaluation setup. 5Greplay and the proposed methodology were applied to all four target cores, whereas ASNFuzzGen was applied only to Open5GS and OAI because its instrumentation environment supports C/C++-based implementations. For each evaluated core, 5Greplay used 7103 test cases, ASNFuzzGen used 8288 test cases for each of the two supported cores, and the proposed methodology used 324 attack scenarios.

Experimental procedure. The comparison was conducted using the same hardware and network environment as the first experiment. Each tool was applied to its respective target cores specified above while preserving the input-generation method and execution procedure provided by its public implementation. The time required to generate and transmit the test cases and observe the resulting behavior was measured.

Analysis method. The comparison considered the number of test cases generated by each method, the number of NGAP message types effectively tested, the average execution time per test, and the range of AMF connection states in which each method could execute tests. These metrics characterize differences in input-generation scale, message range, execution characteristics, and state-dependent testing scope. The comparison was not conducted under an equal time budget and therefore does not measure vulnerability-discovery efficiency or coverage growth over time.

### 4.4. Evaluation Metrics

Six evaluation metrics are used to analyze the two experiments, as summarized in Table 8. Vulnerability count and interpretable outcome rate are used in the first experiment to characterize the security-verification results and the observability of AMF processing outcomes. Test-case count, message coverage, average time per test, and state coverage are used in the second experiment to compare the testing scope and execution characteristics of the proposed methodology and the existing NGAP fuzzing tools.

Vulnerability count. The vulnerability count is the number of scenarios whose observed processing outcomes differ from the specification-defined expectations and are confirmed through further analysis to constitute security vulnerabilities. Missing or abnormal responses are not automatically counted as vulnerabilities. Only cases in which analysis of the NGAP processing outcome and core logs confirms both a security-requirement violation and a security impact are included.

Interpretable outcome rate. The interpretable outcome rate represents the proportion of executed test scenarios for which the AMF processing outcome can be determined from an explicit NGAP response or sufficient processing logs. A test scenario is classified as interpretable only when the available evidence is sufficient to determine how the AMF processed the test input. This metric therefore reflects the observability and interpretability of AMF processing outcomes rather than the overall effectiveness of the testing methodology:

(1) $$R_{\mathrm{interpretable}}=\frac{N_{\mathrm{interpretable}}}{N_{\mathrm{total}}}\times 100,$$

where $N_{\mathrm{interpretable}}$ is the number of executed test scenarios for which the AMF processing outcome can be determined, and $N_{\mathrm{total}}$ is the total number of executed test scenarios.

Test-case count. The test-case count is the total number of test cases configured or generated by each method for a single core. Different mutations applied to the same message are counted as separate test cases. The same mutation applied in different connection states is also counted separately.

Message coverage. Message coverage is defined as the number of uplink NGAP message types effectively tested by each method among the 40 uplink message types considered in this study. A message type is counted only once, even when multiple test cases are applied to the same message. Messages that do not reach the AMF are excluded.

Average time per test. The average time per test is calculated by dividing the total execution time by the total number of executed test cases:

(2) $$T_{\mathrm{avg}}=\frac{T_{\mathrm{total}}}{N_{\mathrm{total}}},$$

where $T_{\mathrm{total}}$ is the total time required to execute all test cases, and $N_{\mathrm{total}}$ is the number of executed test cases. The total execution time includes test-case generation, connection-state establishment where applicable, mutated-message transmission, and outcome observation. The average time per test is reported in seconds.

State coverage. State coverage represents the proportion of the defined AMF connection states in which each method can execute tests:

(3) $$R_{\mathrm{state}}=\frac{N_{\mathrm{covered}}}{N_{\mathrm{state}}}\times 100,$$

where $N_{\mathrm{covered}}$ is the number of connection states in which the method can execute tests, and $N_{\mathrm{state}}$ is the total number of connection states defined in this study.

## 5. Experimental Results

### 5.1. Security Verification Results for 5G AMFs

#### 5.1.1. Interpretable Outcome Rate by Mutation Type

A total of 1296 tests were conducted by applying 324 attack scenarios to each of the four target AMFs. Table 9 summarizes the interpretable outcomes by mutation type and core implementation.

An interpretable AMF processing outcome was obtained in 877 of the 1296 tests, yielding an overall interpretable outcome rate of 67.7%. Open5GS produced the largest number of interpretable outcomes, with 233 cases, followed by ella-core with 222, free5GC with 216, and OpenAirInterface with 206.

Among the mutation types, mandatory IE omission achieved the highest interpretable outcome rate at 71.7%, followed by identifier mismatch at 69.3% and state violation at 67.8%. When a mandatory IE was removed, the remaining message structure was preserved, allowing the input to reach the mandatory-IE validation stage. Identifier mismatch and state violation likewise preserved the ASN.1 structure, enabling the messages to reach post-decoding logic that validates UE identifiers, UE contexts, and connection states.

Syntactic and semantic mutations showed lower interpretable outcome rates of 66.8% and 58.1%, respectively. Syntactic mutations violated IE data types or permitted value ranges, causing some inputs to be rejected during ASN.1 decoding or preliminary constraint validation. Semantic mutations remained syntactically valid but contained procedurally invalid values or inconsistent IE combinations. In some cases, processing was terminated during procedure-specific validation without producing an explicit response or sufficient processing log. Security capability mutation consisted of a single scenario, and an observable processing outcome was obtained from all four cores, resulting in an interpretable outcome rate of 100%.

The number of interpretable outcomes ranged from 206 to 233 across the four cores. This relatively narrow range indicates that the same state-establishment and mutated-message transmission procedure could be applied across independently developed core implementations. The remaining 419 scenarios (32.3%) did not provide sufficient evidence for a definitive interpretation of the AMF processing outcome. Of these, 238 scenarios (18.4% of all executed scenarios) were rejected during encoding or decoding, primarily because the mutated inputs violated ASN.1 structural, type, or constraint requirements before reaching the intended NGAP processing logic. Another 119 scenarios (9.2%) produced no explicit AMF response after transmission, making it difficult to distinguish defensive silent rejection from internal processing that did not generate a protocol-level response. The remaining 62 scenarios (4.8%) did not provide sufficient processing logs to reliably determine the final AMF behavior; this lack of logging also represents a limitation in the observability and auditability of the corresponding processing outcomes. These categories are reported separately because they represent distinct causes of outcome unavailability rather than a single measure of test effectiveness. Specification-compliance and vulnerability classification were performed only on the 877 scenarios for which the AMF processing outcome could be interpreted with sufficient confidence, while all 1296 scenarios were retained in the analysis of outcome distribution and observability.

#### 5.1.2. Interpretable Outcome Rate by AMF State

**Figure 7:**
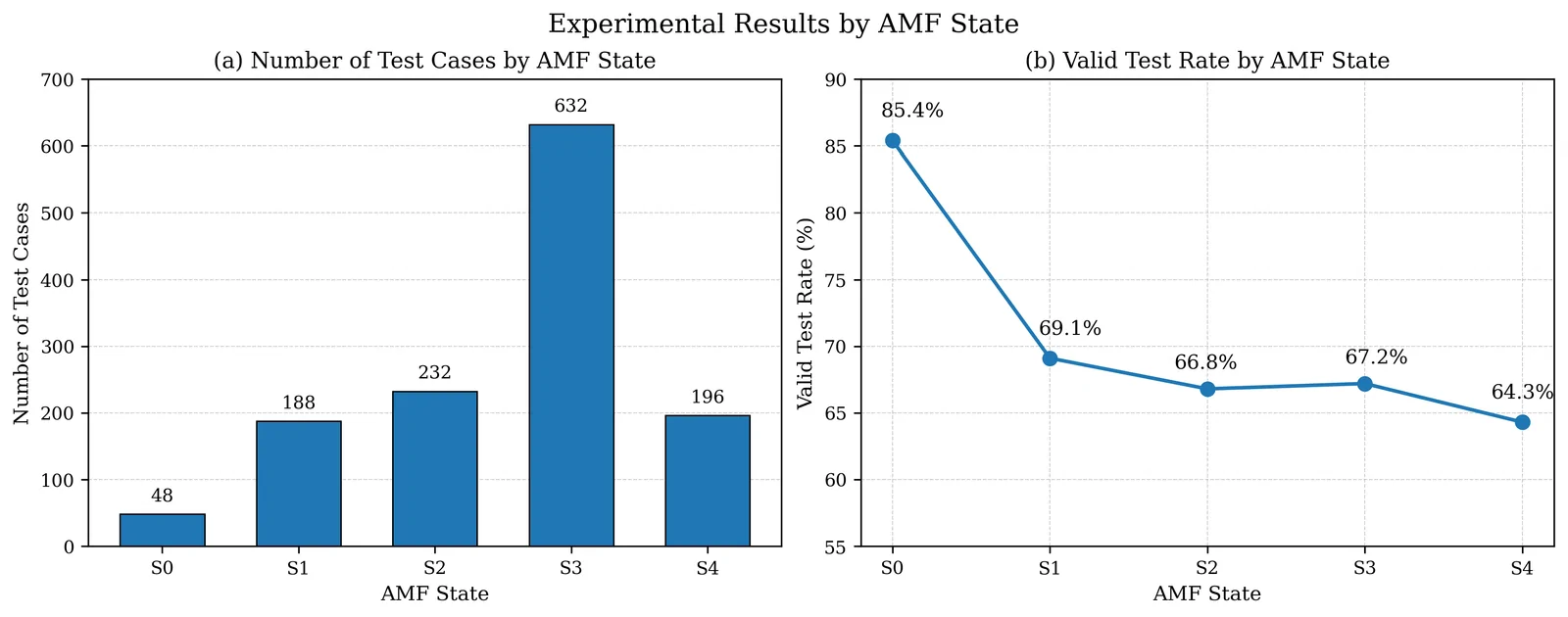
Test-case count and interpretable outcome rate by AMF connection state.

The state-based analysis showed that 632 of the 1296 tests targeted S3, followed by 232 tests in S2 and 196 tests in S4. This distribution reflects the identical state-specific scenario configuration applied to each of the four cores. Figure 7 presents the number of test cases and the interpretable outcome rate for each AMF state.

The interpretable outcome rate was highest in S0 at 85.4%, whereas the rates for S1 through S4 ranged from 64.3% to 69.1%. In S0, mutated messages could be transmitted without executing preceding NGAP procedures, which reduced the number of state-establishment steps that could prevent subsequent outcome interpretation. In states beyond S0, normal NGAP procedures had to be completed before the target state could be reached. Consequently, some scenarios did not proceed to mutated-message transmission because the required state transition was not completed, while others produced no explicit response or sufficient processing logs after transmission.

Among the 1060 tests performed in S2 or later states, 706 produced interpretable outcomes, corresponding to an interpretable outcome rate of 66.6%. This value was 1.1 percentage points lower than the overall rate of 67.7%. The rates for S2 and S3 were similar, being 66.8% and 67.2%, respectively, and no continuous decrease was observed as the connection state progressed. These results indicate that deeper connection states did not necessarily reduce outcome interpretability, although additional state-establishment and procedure-dependent processing introduced more opportunities for the processing outcome to become unavailable for interpretation.

S4 exhibited the lowest interpretable outcome rate at 64.3%. In some core implementations, the Handover Preparation procedure did not complete successfully, preventing execution of the subsequent mutated-message test. In other cases, the mutated message was transmitted but produced neither an explicit response nor sufficient processing-log information. These scenarios were not treated as ineffective tests; instead, they were recorded separately according to the cause that prevented reliable interpretation of the AMF processing outcome.

#### 5.1.3. Vulnerability Analysis

The observed outcomes of the 877 interpretable scenarios were compared with the specification-defined expected behaviors. This analysis identified 32 specification violations. Among these violations, 11 cases resulted in security-relevant effects and were classified as security vulnerabilities. Results repeatedly observed across multiple scenarios but caused by the same underlying flaw were consolidated into a single case to avoid duplicate counting. Table 10 presents the distribution of specification violations by mutation type and core implementation.

The number of specification violations was 10 for ella-core, 9 for Open5GS, 7 for free5GC, and 6 for OpenAirInterface. Identifier mismatch produced the largest number of violations, with seven cases. Mandatory IE omission, semantic mutation, and state violation each produced six cases. Security capability mutation produced four cases, and syntactic mutation produced three.

The six violations associated with mandatory IE omission occurred when the AMF failed to perform the error-handling behavior required for a message missing a mandatory IE. The three syntactic-mutation violations fell into two categories: inputs that violated IE data types or permitted ranges but were propagated beyond decoding, and inputs for which the required error response was not returned. The six semantic-mutation violations involved syntactically valid but procedurally invalid values or inconsistent IE combinations that were not explicitly rejected by the AMF. Although these behaviors differed from the specification-defined expectations, no direct security impact, such as alteration of a UE identifier or security context, was confirmed.

**Figure 8:**
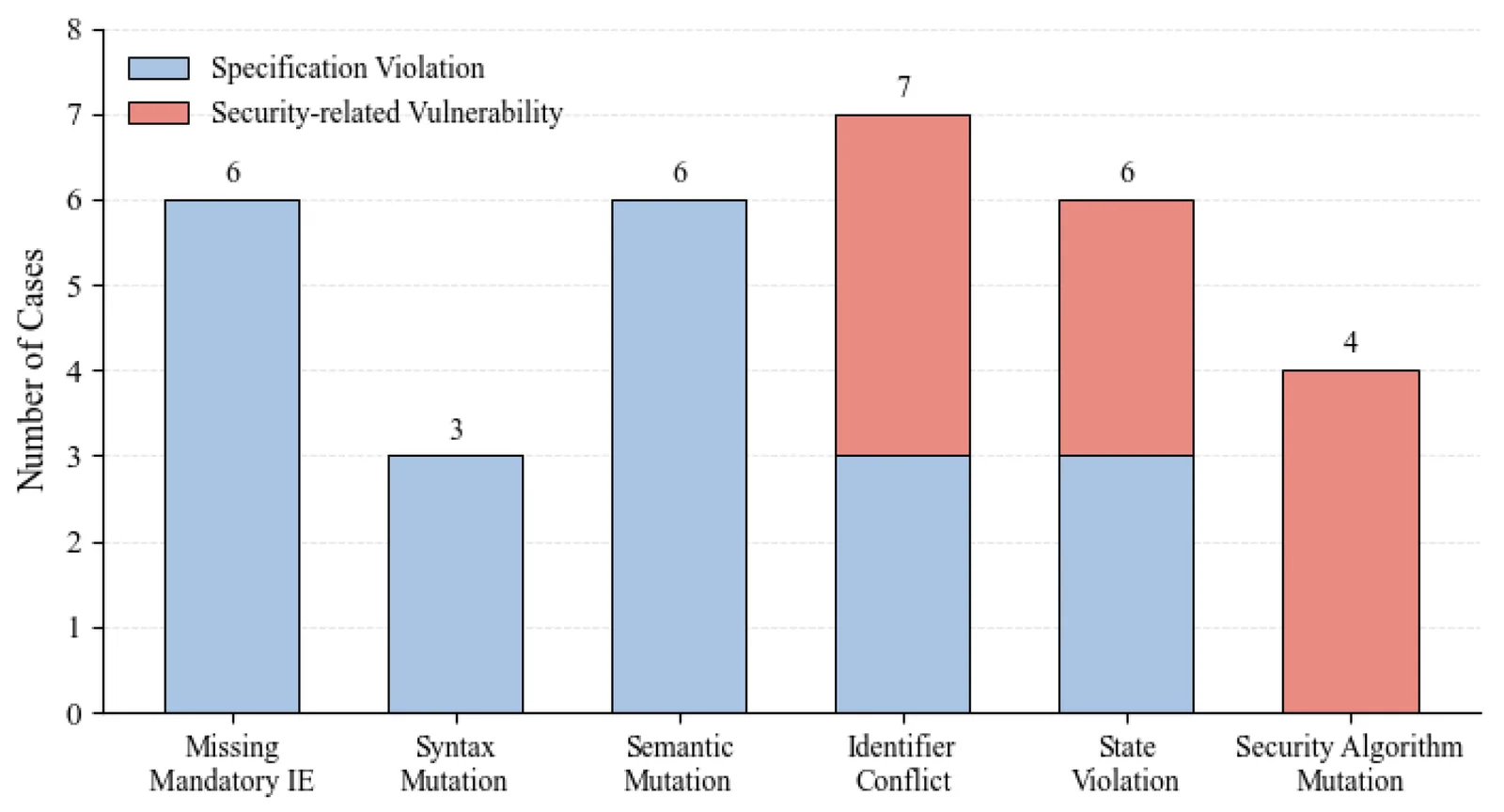
Distribution of specification violations and security vulnerabilities by mutation type.

Figure 8 presents the distribution of specification violations and the subset classified as security vulnerabilities.

Security impact was confirmed in 11 of the 32 violations, consisting of four identifier-mismatch cases, three state-violation cases, and four security-capability-mutation cases. By contrast, the violations associated with mandatory IE omission, syntactic mutation, and semantic mutation involved missing error handling or procedure-specific validation inconsistencies, but no direct security impact was confirmed. These vulnerabilities should be interpreted under the attacker assumptions defined in Section 3.1. They do not imply exploitability by an arbitrary unauthenticated remote attacker, because reaching the tested states and transmitting the corresponding NGAP messages requires N2 access and gNB-level protocol capabilities. Cases in which only specification non-compliance or abnormal error handling was observed without a confirmed security-relevant effect are therefore reported as specification violations rather than security vulnerabilities.

Identifier-mismatch vulnerabilities occurred when an AMF accepted a message containing UE identifiers that did not match the current NGAP connection, potentially allowing the message to reference or modify another UE context. State-violation vulnerabilities occurred when a subsequent NGAP procedure was executed despite the absence of the required UE context or preceding procedure. Security-capability-mutation vulnerabilities occurred when the AMF incorporated the UE Security Capabilitiesreceived in a Path Switch Request into the UE context without comparing them with the previously stored values. Consequently, mutated security-algorithm information could alter the existing UE security capabilities.

The confirmed vulnerabilities were reported to the corresponding open-source projects. Following developer review, the affected processing logic was revised to validate the consistency between UE identifiers and connection information, verify the UE context and state required for each procedure, and compare newly received UE security capabilities with the previously stored values.

All identified vulnerabilities occurred while the affected cores remained operational. No crash, abnormal termination, or memory error was observed during testing. Instead, the AMFs processed specification-violating inputs as part of normal NGAP procedures or modified security-related context information without performing the required validation.

### 5.2. Comparison with Existing NGAP Fuzzing Tools

#### 5.2.1. Test-Case Count and Message Coverage

**Figure 9:**
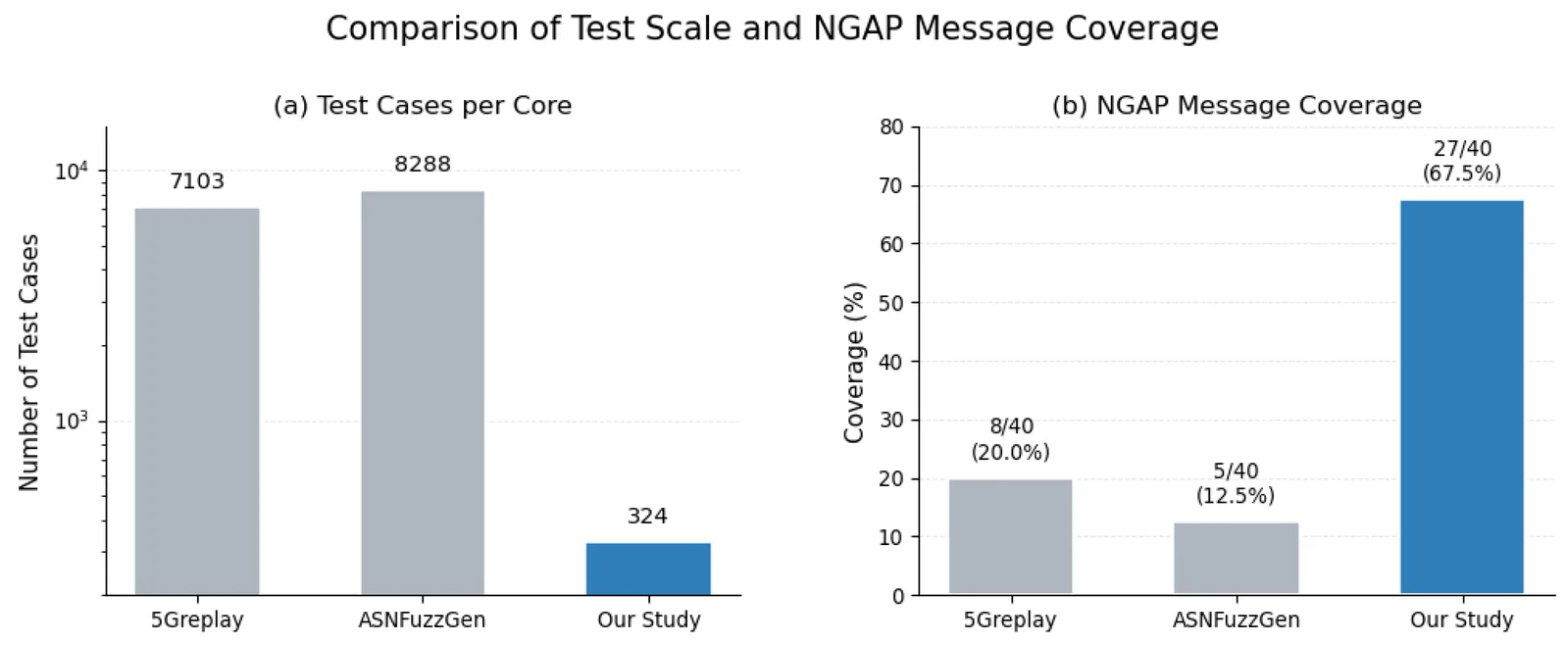
Test-case count and NGAP message coverage of the compared methods.

5Greplay generated 7103 test cases for each of the four target cores, whereas ASNFuzzGen generated 8288 test cases for each of the two supported C/C++-based cores, Open5GS and OAI. The proposed methodology executed 324 attack scenarios for each of the four target cores. Despite generating fewer test cases, the proposed methodology tested 27 of the 40 uplink NGAP message types, corresponding to 67.5% coverage. In comparison, 5Greplay tested 8 message types, corresponding to 20.0%, and ASNFuzzGen tested 5 message types, corresponding to 12.5%. Figure 9 compares the test-case count and NGAP message coverage of the three methods.

5Greplay and ASNFuzzGen generated large numbers of test cases through repeated mutation of collected messages and ASN.1 schema-based input generation, respectively. However, the testing scope of 5Greplay depended on the messages available in the collected traffic, whereas ASNFuzzGen generated individual ASN.1 messages without transitioning the AMF through the required connection states. These approaches increased the number of test cases by applying multiple mutations to the same message, but they did not readily cover NGAP messages that required preceding procedures.

The proposed methodology constructed attack scenarios from combinations of messages applicable to each connection state and the defined mutation types, rather than prioritizing large-scale input generation. Consequently, it generated fewer test cases but exercised session-management and mobility-management messages that become available only after the establishment of the UE-associated logical NG connection and UE context. Whereas the existing tools applied many inputs to a limited set of messages, the proposed methodology restricted the input volume while broadening the range of tested NGAP messages.

These results indicate a trade-off between input volume and testing scope rather than an overall efficiency advantage of any method. 5Greplay and ASNFuzzGen generated larger numbers of inputs, whereas the proposed methodology used a smaller, state-dependent scenario set covering 27 NGAP message types across different procedures. Because the methods employ different input-generation strategies and were not evaluated under an equal time budget, the observed message coverage should be interpreted as the scope reached by each configured test set rather than as a coverage-growth or discovery-efficiency comparison.

#### 5.2.2. Execution Time and State Coverage

**Figure 10:**
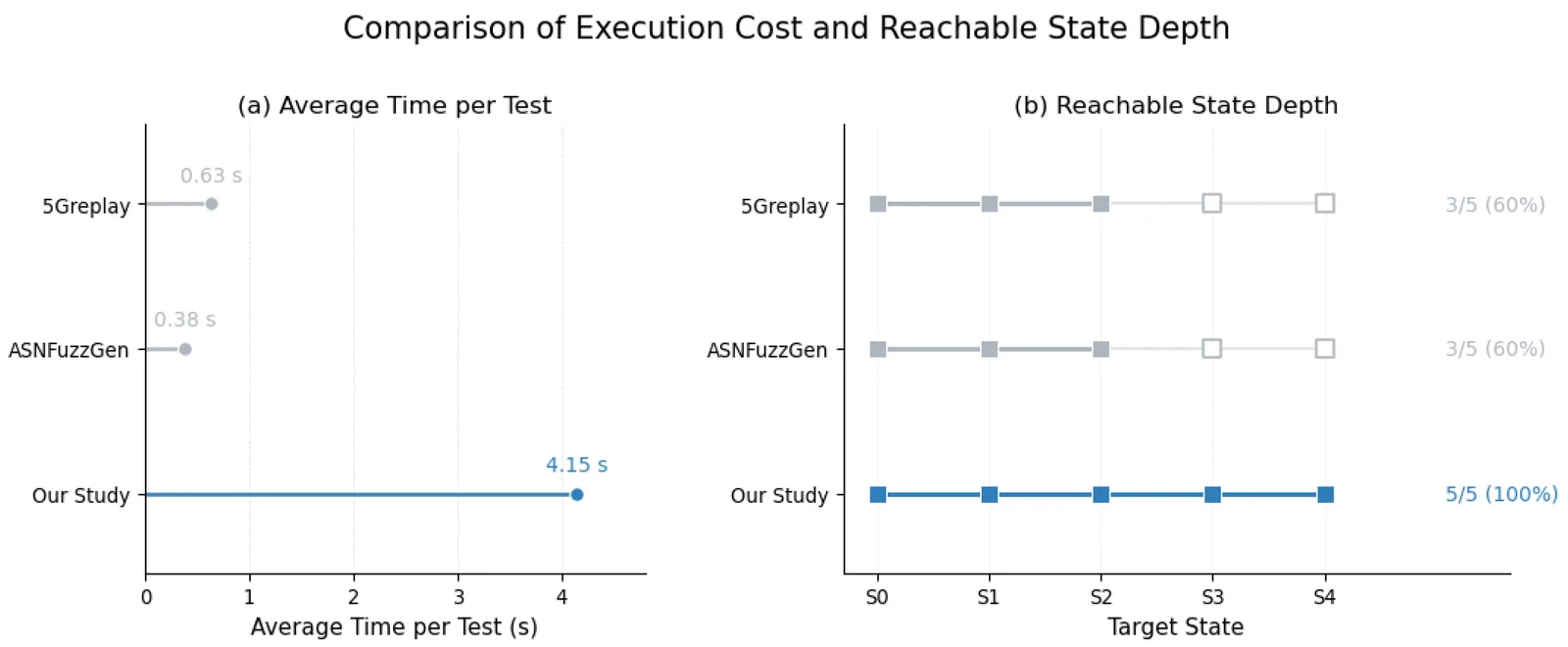
Average time per test and AMF state coverage of the compared methods.

The average time per test was 0.63 s for 5Greplay, 0.38 s for ASNFuzzGen, and 4.15 s for the proposed methodology. Both 5Greplay and ASNFuzzGen performed tests in three states, from S0 to S2, resulting in state coverage of 60%. The proposed methodology performed tests in all five states, from S0 to S4, achieving state coverage of 100%. Figure 10 compares the average execution time and state coverage of the three methods.

5Greplay and ASNFuzzGen continuously replayed or generated individual inputs without executing the preceding procedures required to establish a target state. Their average execution times were therefore shorter, but they could not intentionally establish S3, in which the UE context had been established, or S4, in which Handover Preparation had been completed. Reaching these states required the sequential completion of normal NGAP procedures, including Initial Context Setup and Handover Preparation.

The proposed methodology established the state required by each scenario before transmitting the mutated message and reset the connection and UE context after the test. These steps increased the average time per test but allowed messages to be transmitted in all states from S0 to S4. Accordingly, the average time of 4.15 s included not only input generation and transmission but also target-state establishment, observation of the mutated-message processing outcome, and initialization for the subsequent scenario.

The execution-time and state-coverage results reflect a trade-off between testing speed and state-dependent testing scope. Existing tools transmitted individual inputs more quickly, whereas the proposed methodology incurred additional overhead for state establishment, outcome observation, and initialization while extending the testing scope to S3 and S4. The average execution time therefore characterizes the additional cost of state-dependent testing and should not be interpreted as a measure of vulnerability-discovery efficiency. Since vulnerability discovery under an equal time budget was not evaluated, no claim is made that the proposed methodology is more efficient than the comparison tools in this respect.

## 6. Conclusions

This study proposed a stateful NGAP security verification methodology for 5G core implementations by combining stateful fuzzing with specification-guided security verification. We analyzed NGAP procedures and their dependencies in the 3GPP specifications to define a connection-state model from S0 to S4. We then constructed 324 attack scenarios by combining state-specific target messages, mutation types, and expected behaviors. For each scenario, the required connection state was established through a sequence of normal NGAP procedures, after which a mutated message was transmitted. The resulting AMF responses and processing logs were compared with the specification-defined expected behavior.

A total of 1296 tests were conducted across four open-source 5G core implementations. An interpretable AMF processing outcome was obtained in 877 tests, yielding an interpretable outcome rate of 67.7%. 5Greplay and ASNFuzzGen tested 8 and 5 uplink NGAP message types, respectively, across states S0–S2. In contrast, the proposed methodology tested 27 of the 40 uplink NGAP message types across all five states from S0 to S4. These results show that the proposed methodology extends the testing scope to procedures that require an established UE context or an ongoing handover procedure.

The comparison of the observed processing outcomes with the specification-defined expectations identified 32 specification violations, including 11 security vulnerabilities. The vulnerabilities involved accepting UE identifiers that were inconsistent with the current connection, executing subsequent procedures without the required UE context, and updating stored UE security capabilities without the required validation. None of the identified vulnerabilities caused a core to crash or produced a memory error. Instead, they occurred when an AMF processed a specification-violating input as part of a normal NGAP procedure. These findings demonstrate that NGAP security verification should consider both the connection state in which a message is received and the processing behavior required by the specifications.

The proposed methodology has several limitations. First, the evaluation was limited to uplink NGAP messages transmitted from the gNB to the AMF and did not examine downlink NGAP messages. Second, the state model was derived from the NGAP procedures included in the evaluation and therefore does not represent every internal AMF state or procedure. Third, the processing outcomes of some scenarios could not be determined because no explicit AMF response or sufficient processing log was observed. Fourth, exploitability under weaker attacker models, such as attackers without N2 access or gNB-level protocol capabilities, was not experimentally evaluated. Accordingly, the identified vulnerabilities should not be interpreted as demonstrating unauthenticated remote exploitability.

The current evaluation also does not consider continuous anomaly injection on top of abnormal states created by failed, interrupted, or recovery procedures. The state-violation scenarios in this study inject messages that are invalid for a normally established target state, but they do not use an abnormal or partially recovered state itself as the starting condition for additional mutated inputs. Extending the methodology to incorporate such abnormal transitions and recovery paths would enable more comprehensive exploration of the NGAP state space.

Although the current evaluation uses uplink NGAP messages transmitted from the gNB to the AMF as test inputs, the observed processing outcomes already include AMF responses and subsequent downlink NGAP messages. The proposed methodology can therefore be extended to a bidirectional NGAP workflow by treating an uplink input and its corresponding downlink responses or subsequent procedure messages as a single state-dependent NGAP sequence. For each sequence, the specification-defined message flow, expected responses, and state transitions can be compared with the observed procedure. This extension would allow the verification to assess not only whether an uplink input is accepted or rejected, but also whether the resulting NGAP procedure follows the specification-defined bidirectional workflow.

The outcome-analysis process should also be improved to infer processing results from subsequent NGAP messages and connection-state changes when no explicit response is returned.

## Figures and Tables

**Table 1:** Comparison of related studies.

Study	Protocol			Technique			
	NGAP	NAS	RRC	Mutation-Based	Structure-Aware	Stateful	Specification-Guided
5Greplay [4]	•	•	○	•	○	○	○
AMFuzz [5]	•	•	○	•	○	○	○
ASNFuzzGen [9]	•	•	○	•	•	○	○
Hu et al. [6]	•	○	○	•	•	○	○
Hsu et al. [7]	•	•	○	•	○	•	○
5GC-Fuzz [8]	•	•	○	•	•	•	○
CoreCrisis [14]	○	•	○	•	○	•	•
CITesting [13]	○	•	○	○	○	•	•
LTEFuzz [10]	○	•	•	○	○	○	•
5GReasoner [11]	○	•	•	○	○	•	•
DoLTEst [12]	○	•	•	○	○	•	•
This work	•	○	○	•	○	•	•

• indicates that the study includes the corresponding protocol or technique, and ○ indicates that the item is not included. Mutation-based denotes the generation of test inputs by modifying valid protocol messages. Specification-guided denotes the use of specification-derived requirements or expected behaviors to evaluate test outcomes.

**Table 2:** Literature-based comparison of NGAP fuzzing approaches.

Study	Test Construction	Use of State/Context	Evaluation Criterion
5Greplay [4]	Replay and mutation of captured traffic	No explicit state model	Abnormal responses and service impact
AMFuzz [5]	Mutation of valid NGAP messages	No explicit state model	Crashes and abnormal behavior
Hu et al. [6]	Weighted mutation of NGAP fields	No explicit state model	Processing behavior of mutated inputs
Hsu et al. [7]	Mutation during ongoing procedures	Procedure context preserved	Abnormal processing and state behavior
5GC-Fuzz [8]	State-guided input generation	Protocol state machine	Crashes and abnormal state transitions
ASNFuzzGen [9]	ASN.1 structure-aware generation	No explicit state model	Execution failures and implementation defects
This work	State-dependent mutation scenarios	NGAP connection and UE-context states	Specification-defined expected behavior

**Table 3:** AMF connection states and representative NGAP messages applicable to each state.

State	Description	Representative Messages
S0	An SCTP connection exists, but the NG connection between the gNB and AMF has not been established	NG Setup Request
S1	The NG connection has been established, but no UE-associated logical NG connection exists	Initial UE Message
S2	A UE-associated logical NG connection exists, but the UE context has not been established	Initial Context Setup Response, Uplink NAS Transport
S3	The UE context has been established, allowing most UE-associated procedures to be performed	Handover Required, Path Switch Request, PDU Session Resource Setup Response
S4	A preceding handover-related procedure is in progress	Handover Cancel, Handover Notify

**Table 4:** NGAP mutation types, application methods, specification basis, and expected AMF behaviors.

Mutation Type	Application Method	Specification Basis	Expected Behavior
Mandatory IE omission	Remove a mandatory IE	TS 38.413, Section 10.3.5	A missing mandatory IE or IE group shall be handled according to its presence and criticality information.
Syntactic mutation	Assign a value that violates the IE data type or permitted range	TS 38.413, Sections 10.2 and 10.3	Transfer- or abstract-syntax errors, including ASN.1 constraint and logical-range violations, shall be handled according to the specified protocol-error procedures.
Semantic mutation	Assign a syntactically valid value that is not permitted by the procedure	TS 38.413, Section 10.4	A correctly decoded message containing semantically invalid information shall be treated as a logical error and handled using the applicable unsuccessful outcome or Error Indication.
Identifier mismatch	Assign UE identifiers that do not match the current connection	TS 38.413, Section 10.6	An AP ID error shall be handled according to the AP-ID error-handling rules defined for UE-associated signalling.
State violation	Transmit a message that is not permitted in the current connection state	TS 38.413, Section 10.4	A procedure that is incompatible with the receiver state shall be treated as a logical error and handled according to the applicable procedure.
Security capability mutation	Assign UE security capabilities that differ from the stored values	TS 38.413, Section 8.4.4; TS 33.501, Section 6.7.3.1	During the Path Switch procedure, the AMF shall verify the received UE 5G security capabilities against the locally stored capabilities.

**Table 5:** Target cores and experimental environment.

**Target Cores**		
**Core**	**Version**	**Language**
ella-core	v1.8.1	Go
Open5GS	v2.7.6	C
free5GC	v3.3.0	Go
OpenAirInterface (OAI)	v2.0.1	C/C++
**Execution Environment**		
Host	8 vCPUs, 16 GB RAM	
Operating system	Ubuntu 22.04.5 LTS, kernel 6.8.0	
RAN/UE	UERANSIM v3.2.7	

**Table 6:** Distribution of attack scenarios by mutation type.

Mutation Type	Number of Scenarios	Proportion
Mandatory IE omission	76	23.5%
Syntactic mutation	55	17.0%
Semantic mutation	40	12.3%
Identifier mismatch	44	13.6%
State violation	108	33.3%
Security capability mutation	1	0.3%
Total	324	100%

**Table 7:** Distribution of attack scenarios by AMF connection state.

Connection State	Number of Scenarios	Proportion
S0	12	3.7%
S1	47	14.5%
S2	58	17.9%
S3	158	48.8%
S4	49	15.1%
Total	324	100%

**Table 8:** Evaluation metrics used in the two experiments.

Metric	Experiment	Description
Vulnerability count	First	Number of scenarios confirmed as security vulnerabilities
Interpretable outcome rate	First	Proportion of executed test scenarios for which the AMF processing outcome could be determined (%)
Test-case count	Second	Number of test inputs transmitted by each method to one core
Message coverage	Second	Number of effectively tested message types among the 40 uplink NGAP message types
Average time per test	Second	Total execution time divided by the number of executed test cases (s)
State coverage	Second	Proportion of defined AMF connection states in which testing can be performed (%)

**Table 9:** Interpretable outcomes by mutation type.

Mutation Type	Interpretable Outcomes/Test Cases				Total	
	Ella-Core	Open5GS	Free5GC	OAI	Interpretable Outcomes	Interpretable Outcome Rate
Mandatory IE omission	55/76	60/76	52/76	51/76	218/304	71.7%
Syntactic mutation	37/55	40/55	36/55	34/55	147/220	66.8%
Semantic mutation	23/40	26/40	22/40	22/40	93/160	58.1%
Identifier mismatch	30/44	32/44	32/44	28/44	122/176	69.3%
State violation	76/108	74/108	73/108	70/108	293/432	67.8%
Security capability mutation	1/1	1/1	1/1	1/1	4/4	100%
Total	222/324	233/324	216/324	206/324	877/1296	67.7%

**Table 10:** Distribution of specification violations across open-source 5G cores.

Mutation Type	Ella-Core	Open5GS	Free5GC	OAI	Total
Mandatory IE omission	3	1	1	1	6
Syntactic mutation	0	2	1	0	3
Semantic mutation	3	1	2	0	6
Identifier mismatch	2	2	1	2	7
State violation	1	2	1	2	6
Security capability mutation	1	1	1	1	4
Total	10	9	7	6	32

## Data Availability

The data are contained within the article.

## References

[1] 3GPP (2025). NG-RAN; NG Application Protocol (NGAP).

[2] 3GPP (2024). System Architecture for the 5G System (5GS).

[3] 3GPP (2025). Security Architecture and Procedures for 5G System.

[4] Salazar Z., Nguyen H.N., Mallouli W., Cavalli A.R., Montes de Oca E. (2021). 5Greplay: A 5G network traffic fuzzer—Application to attack injection. Proceedings of the 16th International Conference on Availability, Reliability and Security (ARES), Vienna, Austria, 17–20 August 2021.

[5] Mancini F., Da Canal S., Bianchi G. (2024). AMFuzz: Black-box fuzzing of 5G core networks. Proceedings of the 19th Wireless On-Demand Network Systems and Services Conference (WONS), Chamonix, France, 29–31 January 2024.

[6] Hu Y., Yang W., Cui B., Zhou X., Mao Z., Wang Y. (2021). Fuzzing method based on selection mutation of partition weight table for 5G core network NGAP protocol. Innovative Mobile and Internet Services in Ubiquitous Computing.

[7] Hsu J.-W., Jiang X.-Y., Chen I.-W., Chen K.-J., Ou-Yang C., Huang C.-Y. (2025). Toward a robust ingress for open-sourced 5G core network. IEEE Trans. Reliab..

[8] Sun Y., Liu X., Sun Q., Wang J., Tian L., Liu J. (2025). 5GC-Fuzz: Finding deep stateful vulnerabilities in 5G core network with black-box fuzzing. Proceedings of the IEEE International Conference on Computer Communications (INFOCOM), London, UK, 19–22 May 2025.

[9] Bennett N., Zhu W., Simon B., Kennedy R., Enck W., Traynor P., Reaves B. (2024). RANsacked: A domain-informed approach for fuzzing LTE and 5G RAN-core interfaces. Proceedings of the ACM SIGSAC Conference on Computer and Communications Security (CCS), Salt Lake City, UT, USA, 14–18 October 2024.

[10] Kim H., Lee J., Lee E., Kim Y. (2019). Touching the untouchables: Dynamic security analysis of the LTE control plane. Proceedings of the IEEE Symposium on Security and Privacy, San Francisco, CA, USA, 20–22 May 2019.

[11] Hussain S.R., Echeverria M., Karim I., Chowdhury O., Bertino E. (2019). 5GReasoner: A property-directed security and privacy analysis framework for 5G cellular network protocol. Proceedings of the ACM SIGSAC Conference on Computer and Communications Security (CCS), London, UK, 11–15 November 2019.

[12] Park C.J., Bae S., Oh B., Lee J., Lee E., Yun I., Kim Y. (2022). DoLTEst: In-depth downlink negative testing framework for LTE devices. Proceedings of the 31st USENIX Security Symposium, Boston, MA, USA, 10–12 August 2022.

[13] Son M., Kim K., Oh B., Kim Y. CITesting: Systematic testing of context integrity violations in LTE core networks. Proceedings of the ACM SIGSAC Conference on Computer and Communications Security (CCS).

[14] Dong Y., Yang T., Al Ishtiaq A., Rashid S.M.M., Ranjbar A., Tu K., Wu T., Mahmud M.S., Hussain S.R. CoreCrisis: Threat-guided and context-aware iterative learning and fuzzing of 5G core networks. Proceedings of the 34th USENIX Security Symposium.

[15] Tang Q., Ermis O., Nguyen C.D., De Oliveira A., Hirtzig A. (2022). A systematic analysis of 5G networks with a focus on 5G core security. IEEE Access.

[16] Dumitru-Guzu M., Vlădeanu C. (2024). A vulnerability assessment of open-source implementations of fifth-generation core network functions. Future Internet.

[17] 3GPP (2024). Non-Access-Stratum (NAS) Protocol for 5G System (5GS).

[18] Silveira L.B.D., de Resende H.C., Both C.B., Marquez-Barja J.M., Silvestre B.O., Cardoso K.V. (2022). Tutorial on communication between access networks and the 5G core. Comput. Netw..

[19] Ella Networks Ella Core: Open-Source Mobile Core for Private Networks. https://github.com/ellanetworks/core.

[20] Open5GS Open5GS: Open-Source Implementation for 5G Core and EPC. https://open5gs.org.

[21] Free5GC Free5GC: Open-Source 5G Core Network. https://free5gc.org.

[22] OpenAirInterface Software Alliance. OAI Core Network. https://openairinterface.org/core-network/.

[23] Güngör A. (2021). UERANSIM: Open Source 5G UE and RAN (gNodeB) Implementation. https://github.com/aligungr/UERANSIM.

